# Duration of Folic Acid Supplementation and Adverse Pregnancy Outcomes: A Prospective Multicenter Cohort Study in China

**DOI:** 10.3390/nu18010081

**Published:** 2025-12-26

**Authors:** Mingxuan Zhang, Hongzhao Yu, Hongtian Li, Yubo Zhou, Jianmeng Liu

**Affiliations:** 1 Institute of Reproductive and Child Health/National Health Commission Key Laboratory of Reproductive Health, Peking University Health Science Center, 38 Xueyuan Rd., Beijing 100191, China; 2211110186@stu.pku.edu.cn (M.Z.); bright@bjmu.edu.cn (H.Y.); liht@bjmu.edu.cn (H.L.); 2 Department of Epidemiology and Biostatistics, School of Public Health, Peking University Health Science Center, 38 Xueyuan Rd., Beijing 100191, China; 3 State Key Laboratory of Female Fertility Promotion, Peking University Third Hospital, Beijing 100191, China

**Keywords:** advanced maternal age, folic acid, maternal nutrients, pregnancy outcomes, pre-pregnancy BMI

## Abstract

**Background:** Folic acid supplementation (FAS) before and in early pregnancy prevents neural tube defects, but the benefits of extending FAS to late pregnancy on pregnancy outcomes remain unclear. We aimed to investigate the associations between duration of FAS and a spectrum of pregnancy outcomes, and to determine whether the associations were modified by maternal age or pre-pregnancy body mass index (BMI). **Methods:** This prospective multicenter study included 15,694 singleton pregnancies. We used mixed-effects log-binomial regression models to estimate the adjusted risk ratios (aRRs) and 95% confidence intervals (CIs) for gestational diabetes mellitus (GDM), gestational hypertensive disorders (GHDs), pre-eclampsia, preterm birth, macrosomia, small (SGA) and large for gestational age (LGA), and the interaction effects of advanced maternal age and pre-pregnancy BMI. **Results**: Of 15,694 women, 4523 (28.8%) did not take FAS before or during pregnancy, 2854 (18.2%) took FAS only during peri-pregnancy, 921 (5.9%) took FAS from peri- to mid-pregnancy, and 7396 (47.1%) took it through late pregnancy. Compared with women without FAS, those supplemented until mid-pregnancy were associated with lower risks of GHDs (aRR 0.84, 95% CI 0.74, 0.96) and pre-eclampsia (aRR 0.81, 95% CI 0.67, 0.97). Supplementation until late pregnancy was associated with lower risks of preterm birth (aRR 0.67, 95% CI 0.59, 0.76), SGA (aRR 0.74, 95% CI 0.63, 0.87), and LGA (aRR 0.88, 95% CI 0.79, 0.97). Among women of advanced maternal age or with overweight/obesity, supplementation until mid-pregnancy was associated with higher risk of GDM. **Conclusions:** Extending FAS until mid-pregnancy is associated with lower risks of GHDs and preeclampsia, and extending it until late pregnancy is associated with lower risks of preterm birth, SGA, and LGA. However, women of advanced maternal age or with overweight/obesity should be cautious about prolonging FAS.

## 1. Introduction

Folate is essential for several interconnected one-carbon metabolism pathways, such as nucleotide biosynthesis, amino acid metabolism, and cellular methylation reactions [1]. To reduce the risk of fetal neural tube defects (NTDs), women planning pregnancy are recommended to take folic acid supplementation (FAS), a synthetic and oxidized form of folate [2]. Women should take 400–800 μg of folic acid (FA) as a daily supplement at least one month before anticipated conception and for the first two to three months of pregnancy [3]. However, FAS beyond the first three months has become increasingly prevalent in recent decades. A cohort study conducted in Hunan province, China (2015), reported that 74% of pregnant women who used folic acid supplements continued its use beyond the first three months [4]. Another Chinese cohort study conducted in Tianjin during 2015–2017 showed that approximately 37% of pregnant women used FAS longer than six months [5]. Moreover, extending FAS during pregnancy is more common in the United States and Canada, with 89% of pregnant women taking FAS until late pregnancy [6,7].

Usage of FAS during the periconceptional period for NTD prevention is well established; however, the potential effects of extending FAS to mid- or late-pregnancy stages remain debatable. Prolonged FAS during pregnancy has been linked, in some animal and human studies, to adverse offspring outcomes including metabolic disorders [8,9] and neurobehavioral alterations [10]. Growing evidence suggests that FAS may reduce adverse pregnancy outcomes by modulating placental development and mitigating oxidative stress [11,12,13,14]. However, emerging evidence also suggests that excessive folate might impair insulin resistance [8,15,16,17] and increase the risk of gestational diabetes mellitus (GDM). Some studies reported that longer FAS duration before or at pregnancy was associated with a higher risk of GDM [4,18,19], while the data on the associations between FAS duration and other pregnancy outcomes, such as preeclampsia [20], are sparse. Only one single-center cohort study on 950 participants has focused on the associations between pre-conceptional FAS duration (longer or less than three months) and multiple pregnancy outcomes, including GDM, cesarean delivery, preterm birth, early-term birth, macrosomia, low birth weight, small for gestational age (SGA), and large for gestational age (LGA) [4]. This study found that using FAS for three months before pregnancy was associated with increased risk of GDM and reduced risk of SGA. Nonetheless, the effects of FAS during pregnancy warrant further investigation. To guide FAS during pregnancy, a thorough understanding of the associations of FAS duration across pregnancy with a spectrum of pregnancy complications and birth outcomes is essential. Additionally, it is also unclear whether these associations vary by maternal age and pre-pregnancy body mass index (BMI). These factors might be related to the folate status [21,22] and have changed drastically with the development of society and fertility policy in China [23,24].

Using a multicenter prospective Chinese cohort data, we aimed to examine the associations between the duration of FAS as well as several pregnancy and birth outcomes. We also sought to investigate whether these associations, if any, varied by mother’s age and pre-pregnancy BMI. Furthermore, we sought to determine whether these associations were modified by maternal age or pre-pregnancy BMI.

## 2. Materials and Methods

### 2.1. Participants and Study Design

Based on the University Hospital Advanced Age Pregnant (UNIHOPE) cohort (ClinicalTrials No.: NCT03220750), our prospective multicenter cohort study was conducted at eight tertiary hospitals across the southern and northern regions of China from March 2017 to June 2021 [25]. Since we aimed to investigate the risk factors of adverse pregnancy outcomes among women with advanced maternal age, women with an anticipated delivery at age ≥ 35 years were primarily recruited. For potential comparisons, we additionally recruited a small proportion of younger females (<35 years at delivery). To be eligible for the UNIHOPE cohort, participants needed to be in early pregnancy, receiving prenatal healthcare, and planning to deliver at the study hospitals. We excluded those participants who did not provide informed consent or exhibited mental disorders. We recruited both singleton and twin pregnancies and used the sub-cohort data of singleton pregnancies.

The participants provided baseline information at enrollment before 14 gestational weeks and completed follow-ups at 24–28 weeks and 32–34 weeks of gestation, after delivery, and 6–12 postpartum weeks, respectively. Trained nurses or obstetricians collected the baseline information on the mother’s age, height, pre-pregnancy body weight, ethnicity, education level, smoking and alcohol consumption within six months before pregnancy, parity, and conception mode using a structured questionnaire. Information on FAS duration, pregnancy complications, and birth outcomes was collected during follow-ups. Using the WHO criterion, we calculated the pre-pregnancy BMI by dividing the pre-pregnancy weight in kilograms by height in meters squared. It was categorized as underweight (<18.5 kg/m^2^), normal-weight (18.5 to <25.0 kg/m^2^), overweight (25 to <30 kg/m^2^), or obese (≥30 kg/m^2^), respectively.

**Figure 1:**
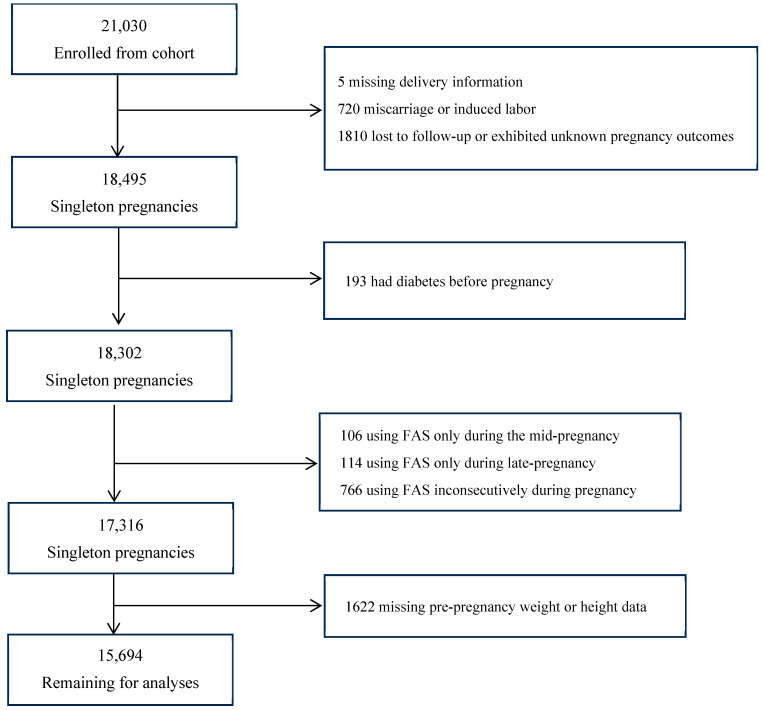
Participant flow chart.

Among our 21,030 singleton pregnant participants, we excluded those with missing delivery information (*n* = 5); who had miscarriage or induced labor (*n* = 720); were lost to follow-up or exhibited unknown pregnancy outcomes (*n* = 1810); had diabetes before pregnancy (*n* = 193); or participants using FAS only during the mid-pregnancy stage (*n* = 106), only during late pregnancy (*n* = 114), or inconsecutively during pregnancy (*n* = 766); or those with missing pre-pregnancy weight or height data (*n* = 1622). Finally, 15,694 participants were analyzed (Figure 1). Maternal and child characteristics were comparable between included (*n* = 15,694) and excluded participants (*n* = 5336) (Table A1).

The study was approved by the Institutional Review Board/Human Subjects Committee of the Peking University Health Science Center (IRB00001052-16009; date of approval: 7 April 2016).

### 2.2. Exposure and Outcomes

Our study’s exposure was FAS duration, which was collected by asking participants whether they were using FAS regularly during the periconceptional period, mid-pregnancy, and late-pregnancy stages, respectively. These supplements included FA tablets, containing 400 μg FA per pill, and multivitamins, which predominantly contain 400 μg or 800 μg FA per pill, depending on the brands [26]. The duration of FAS was classified into no-FAS (no-FAS), FAS only during the periconceptional period (peri-FAS), FAS through the periconceptional period and mid-pregnancy (mid-FAS), and FAS through the periconceptional period, mid-pregnancy, and late-pregnancy (late-FAS) stages, respectively.

Our outcomes of interest included various pregnancy complications (GDM, gestational hypertensive disorders [GHDs], and pre-eclampsia) and birth outcomes (preterm birth, macrosomia, SGA, and LGA). We diagnosed GDM when a 75 g oral glucose tolerance test (OGTT) at 24 to 28 gestational weeks met one of the following three criteria: (1) fasting plasma glucose ≥ 5.1 mmol/L, (2) the 1 h plasma glucose ≥ 10.0 mmol/L, or (3) the 2 h plasma glucose ≥ 8.5 mmol/L [27]. GHDs were defined as new-onset blood pressure ≥ 140/90 mmHg after 20 gestation weeks. Moreover, pre-eclampsia was diagnosed by observing elevated blood pressure and proteinuria after the 20th gestation week [28]. Preterm was defined as delivery occurring before 37 gestational weeks, and macrosomia was interpreted as birth weight ≥ 4000 g. According to the Chinese sex- and gestational week-specific birth weight standards [29], SGA and LGA were defined as birth weight < 10th percentile for gestational age and birth weight > 90th percentile for gestational age, respectively. Since FAS is related to hematological parameters, hemoglobin levels and red blood cell counts at late pregnancy were presented.

### 2.3. Statistical Analyses

Medians (interquartile range, IQR) were used to present continuous variables, which had skewed distributions according to the Kolmogorov–Smirnov tests (*p* < 0.05), and frequencies (%) were used to present categorical variables. Their differences across groups of FAS duration were tested by the Kruskal–Wallis test or chi-square test, as appropriate.

Presenting as risk ratios (RRs) and 95% confidence intervals (95% CIs), the associations between FAS duration and pregnancy outcomes were examined using univariable and multivariable two-level mixed-effect log binomial regression models to account for the potential impact of the north and south regions, which had distinct dietary patterns [30]. The multivariable models were adjusted for the mother’s age, pre-pregnancy BMI, parity, ethnicity, conception mode, education level, pre-pregnancy smoking, and alcohol consumption within six months before pregnancy (Table 1). Evidence suggests offspring sex-dependent effects in associations between maternal FAS and offspring development [31]. Thus, offspring sex was additionally adjusted in sensitivity analysis. Since GDM, GHDs, and preeclampsia were diagnosed during mid-pregnancy, FAS after mid-pregnancy cannot logically influence the risk of these outcomes, and thus mid- and late-FAS groups were combined for the relevant analyses.

In order to investigate whether these associations varied by maternal age and pre-pregnancy BMI, the interactions of FAS duration with maternal age and pre-pregnancy BMI for pregnancy outcomes were assessed by introducing their interaction terms in the regression models. Subsequently, subgroup analyses were conducted, stratified by maternal age (≥35 and <35 years) or pre-pregnancy BMI.

Statistical analyses were performed using R software (version 4.0; R Foundation for Statistical Computing, Vienna, Austria) with a two-sided test. A *p*-value < 0.05 was considered statistically significant.

## 3. Results

### 3.1. Characteristics of Study Participants

Among 15,694 female participants, 4523 (28.8%), 2854 (18.2%), 921 (5.9%), and 7396 (47.1%) were categorized into no-FAS, peri-FAS, mid-FAS, and late-FAS categories, respectively. Table 1 shows the maternal characteristics for FAS duration groups. Women in the late-FAS group were more likely to be older, multipara, conceive naturally, consume alcohol, and were less likely to be overweight/obese before pregnancy, compared with other participants. Women in the late-FAS group had the highest hemoglobin level at late pregnancy (Table A2).

### 3.2. Incidence of Pregnancy Outcomes

The incidence of adverse pregnancy outcomes varied significantly by FAS duration (Table 2). The peri-FAS group displayed the lowest incidence of macrosomia (15.2%), while the mid-FAS group exhibited the highest incidence of GDM (28.2%) and the lowest incidences of GHDs (8.9%) and pre-eclampsia (4.0%). Furthermore, the late-FAS group’s participants displayed the lowest incidences of preterm birth (9.4%) and LGA (17.7%), and the no-FAS group reported the highest incidences of SGA (8.1%), compared with the other groups.

### 3.3. Associations Between FAS Duration and Pregnancy Outcomes

In univariate analyses, FAS during pregnancy displayed lower risks of GHDs, pre-eclampsia, preterm birth, macrosomia, SGA, and LGA; however, mid-FAS was associated with a higher risk of GDM (RR 1.11, 95% CI 1.03, 1.21), compared with the no-FAS group (Table A3). In multivariate analyses with confounder adjustments, the association of mid-FAS with risk of GDM was marginally significant [adjusted risk ratio (aRR) 1.08, 95% CI 0.99, 1.17; *p* = 0.09], whereas its association with lower risks of GHDs (aRR 0.84, 95% CI 0.74, 0.96) and pre-eclampsia (aRR 0.81, 95% CI 0.67, 0.97) persisted (Table 2). The late-FAS group was significantly associated with lower risks of multiple birth outcomes, including preterm birth (aRR 0.67, 95% CI 0.59, 0.76), SGA (aRR 0.74, 95% CI 0.63, 0.87), and LGA (aRR 0.88, 95% CI 0.79, 0.97) (Table 2). The associations largely remained after additionally adjusting for offspring sex (Table A4).

### 3.4. Modification Effects of Maternal Age and Pre-Pregnancy BMI

**Figure 2:**
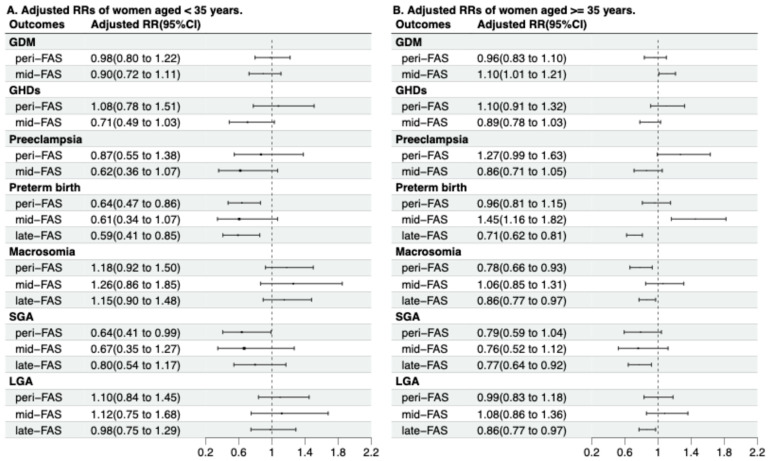
Associations between folic acid supplementation duration and pregnancy outcomes, stratified by maternal age. Abbreviations: FAS—Folic acid supplements; aRRs—adjusted relative risks; GDM—gestational diabetes mellitus; GHDs—gestational hypertensive disorders; SGA—small for gestational age; LGA—large for gestational age. For GDM, GHDs and preeclampsia, which are diagnosed in mid-pregnancy, the late-FAS group was combined with the mid-FAS group for analysis. The analyses for GDM, GHDs, and preeclampsia in the mid-FAS group (which includes late-FAS participants for these mid-pregnancy outcomes) were based on 8317 participants. SGA and LGA analyses included 13,206 participants with complete birth weight and infant sex data. All adjusted RRs (95% CIs) were adjusted for maternal age, pre-pregnancy BMI, parity, ethnicity, conception mode, maternal education, pre-pregnancy smoking, and maternal alcohol consumption.

Maternal age modified the associations of FAS duration with preterm birth and macrosomia (Table A5). The protective effects of peri- and late-FAS on preterm birth were more pronounced in women aged < 35 years (*p*_for interaction_ < 0.01), and on macrosomia were more pronounced in women aged ≥ 35 years (*p*_for interaction_ = 0.03) [Figure 2, Table A5]. In older participants, mid-FAS was associated with a higher risk of GDM (aRR 1.10, 95% CI 1.01, 1.21), though the interaction effects were insignificant (*p*_for interaction_ = 0.14) [Figure 2, Table A5].

**Figure 3:**
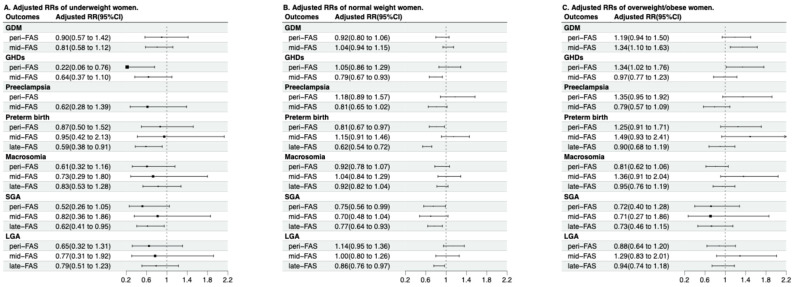
Associations between folic acid supplementation duration and pregnancy outcomes, stratified by pre-pregnancy BMI. Abbreviations: FAS—Folic acid supplements; aRRs—adjusted relative risks; GDM—gestational diabetes mellitus; GHDs—gestational hypertensive disorders; SGA—small for gestational age; LGA—large for gestational age. For GDM, GHDs and preeclampsia, which are diagnosed in mid-pregnancy, the late-FAS group was combined with the mid-FAS group for analysis. The analyses for GDM, GHDs, and preeclampsia in the mid-FAS group (which includes late-FAS participants for these mid-pregnancy outcomes) were based on 8317 participants. SGA and LGA analyses included 13,206 participants with complete birth weight and infant sex data. All adjusted RRs (95% CIs) were adjusted for maternal age, pre-pregnancy BMI, parity, ethnicity, conception mode, maternal education, pre-pregnancy smoking, and maternal alcohol consumption.

Among women with pre-pregnancy overweight/obesity, mid-FAS and peri-FAS exhibited an enhanced risk of GDM (aRR 1.34, 95% CI 1.10, 1.63) (Figure 3), as well as higher risks of GHDs (aRR 1.34, 95% CI 1.02, 1.76; *p*_for interaction_ = 0.01) (Figure 3, Table A5), when compared with the no-FAS group. The associations of FAS duration with GHDs and pre-eclampsia might be modified by pre-pregnancy BMI (Table A5).

## 4. Discussion

In this prospective multicenter study involving 15,694 Chinese participants, peri-FAS subjects displayed a lower risk of macrosomia, while mid-FAS and late-FAS groups were associated with lower risks of GHDs and pre-eclampsia, as well as preterm birth, macrosomia, SGA, and LGA, compared with the no-FAS group, respectively. Moreover, a few associations were modified by maternal age and pre-pregnancy BMI.

We also found that mid-FAS was associated with lower risks of GHDs and pre-eclampsia. When adjusting for FAS duration, meta-analyses showed that FAS versus no-FAS during pregnancy was associated with reduced risk of pre-eclampsia but not GHDs [32,33]. Similarly, when focusing on FAS duration in pregnancy, two cohort studies from the USA and Canada reported that an extended FAS duration was associated with lower the risks of GHDs and pre-eclampsia [34,35]. Our study’s interaction analysis suggested that peri- or mid-FAS might be associated with lower risks of GHDs and pre-eclampsia among underweight or normal weight women, but not in women with overweight/obesity. Consistently, a previous study also reported the association between peri-FAS and lower risk of pre-eclampsia among women with lower BMI, but not in those with higher BMI [36]. Potentially, the extensive metabolic dysregulation in obese women causes enhanced nutrient requirements compared to lean women [37], thereby attenuating or negating any beneficial effects of increased micronutrient intake [38]. Thus, more research is needed to determine the optimum duration of FAS to counteract the metabolic abnormalities in obese or overweight women.

In our study, mid-FAS was associated with a higher risk of GDM, especially in women with advanced maternal age or with pre-pregnancy overweight/obesity. One Chinese cohort study including 1058 pregnant women collected information on FAS during early pregnancy and reported that FAS was associated with a higher risk of GDM (aOR 1.73) [26]; another Chinese study including 326 pregnancies investigated the association between FAS duration during peri-pregnancy and GDM. FAS duration was classified as no-FAS, 1–60 days, 61–90 days, and 91–365 days. This study reported that women using FAS > 90 days during peri-pregnancy displayed a higher risk of GDM than those using FAS ≤ 60 days (aOR 3.45) [19]. Similarly to our findings, a previous Chinese cohort study that included 1938 pregnancies found that, compared to women with a pre-pregnancy BMI < 25 kg/m^2^ who did not take FAS, women with pre-pregnancy overweight/obesity who took FAS daily in early pregnancy exhibited a higher risk of GDM (aOR 5.63) [39]. Since most significant associations between FAS and increased GDM risk were observed among Chinese populations, the effects of ethnic traits and dietary habits need exploration.

We also assessed the effect of late FAS and found that it exhibited lower risks of multiple interested birth outcomes, including preterm birth, SGA, and LGA. Moreover, two meta-analyses concluded that FAS was associated with lower risks of preterm birth and SGA, if initiated before or after conception [40,41]. However, the data regarding LGA and macrosomia were sparse. One Chinese cohort study found that babies of mothers with peri-FAS were heavier than newborns of mothers with no-FAS; this phenomenon was stronger in newborns with lower birth weight [42]. Consistently, FAS was only positively associated with fetal growth without increasing the risk of LGA [43]. Our late-FAS group exhibited lower risks of preterm birth, SGA, and LGA. The associations between late-FAS and fetal growth-related outcomes could be because of the rapid fetal growth in late pregnancy [44].

Though the underlying mechanisms are ambiguous, multiple, non-mutually exclusive pathways might explain the correlation between FAS and pregnancy outcomes. First, as a key nutrient in the one-carbon metabolism cycle, folate lowers the homocysteine concentration, enriches the placental vascular endothelium [11,12], and reduces the risks of GHDs and pre-eclampsia. Serving as a substrate and cofactor, folate is crucial for multiple biochemical processes, such as nucleic acid and protein synthesis, as well as DNA methylation and repair [1]. Exhibiting indispensable effects on the regulation of placental growth and fetal development [13,14], these processes are intimately linked to birth weight and gestational ages [45,46]. A prolonged FAS duration is associated with an increased risk of GDM, possibly because excessive intake of FA could mask vitamin B12 deficiency [47], which exacerbates insulin resistance and causes GDM [48]. Moreover, elevated circulating unmetabolized folic acid (UMFA) levels might promote natural killer cell dysfunction in humans [49], which might be involved in causing insulin resistance and GDM [50]. Notably, the association between extended FAS and GDM risk was primarily observed in Chinese population [4,18,19], possibly since the MTHFR C677T polymorphism was particularly common in this population [51]. The polymorphism can reduce enzyme activity and alter the metabolic response to high-dose or prolonged FA intake, potentially exacerbating insulin resistance through mechanisms involving altered one-carbon metabolism and UMFA accumulation [8,46,48,52]. In addition, maternal obesity is often linked to elevated levels of hepcidin, an index related to chronic inflammation, which could partly explain the adverse effects of FAS observed among women with higher BMI [53].

Our study extended beyond earlier research by involving multiple pregnancy outcomes, including GDM, GHDs, pre-eclampsia, preterm birth, LGA, SGA, and macrosomia, within a large prospective cohort, and by focusing on FAS duration from periconception to late pregnancy. Additionally, we evaluated the modifying effects of the mother’s advanced age and pre-pregnancy BMI. Our study had a few limitations. First, this study used the data exclusively derived from pregnant women from public referral hospitals. This might have introduced selection bias and affected the data extrapolation. Second, because approximately 75% of our participants were of advanced maternal age and 95% were Han Chinese, the generalizability of our findings to younger pregnant women or other ethnic groups may be limited. Third, our results might be biased because we did not collect the data on the FA dose and multivitamins. This introduces the potential for non-differential exposure misclassification, as individuals within the same FAS duration group may have had varying actual daily intakes. Such misclassification might attenuate the observed associations. Additionally, women with advanced maternal age, adverse pregnancy history, or those under medical supervision may have been advised to continue FAS longer, which could bias the association of extended FAS with risk of GDM. Finally, we lacked data on several potential confounders, such as dietary folate, physical activity, and family medical history. Their absence limits causal interpretation, as residual confounding could bias the associations in either direction.

## 5. Conclusions

Extending FAS until mid-pregnancy stage was associated with lower risks of GHDs and pre-eclampsia; however, an enhanced risk of GDM was observed in women with advanced maternal age or overweight/obesity. Moreover, FAS until the late-pregnancy stage was associated with lower risks of multiple adverse birth outcomes, including preterm birth, SGA, and LGA. Thus, prolonged FAS during pregnancy might be beneficial; however, women with advanced maternal age or overweight/obesity should be cautious about extending FAS duration. Additional studies focusing on the effects of FA dosage combined with its duration are needed.

## Figures and Tables

**Table 1:** Characteristics of study participants.

Characteristics	No-FAS (*n* = 4523)	Peri-FAS (*n* = 2854)	Mid-FAS (*n* = 921)	Late-FAS (*n* = 7396)	*p*-Value ^†^
Maternal age, years					
<35	1235 (27.3)	1209 (42.4)	278 (30.2)	1120 (15.1)	<0.01
35–39	2541 (56.2)	1341 (47.0)	501 (54.4)	5079 (68.7)	
40–44	705 (15.6)	289 (10.1)	133 (14.4)	1126 (15.2)	
≥45	42 (0.9)	15 (0.5)	9 (1.0)	71 (1.0)	
Pre-pregnancy BMI, kg/m^2^					
Underweight	389 (8.6)	227 (8.0)	79 (8.6)	652 (8.8)	<0.01
Normal weight	3325 (73.5)	2039 (71.4)	705 (76.5)	5694 (77.0)	
Overweight	650 (14.4)	498 (17.4)	116 (12.6)	937 (12.7)	
Obesity	159 (3.5)	90 (3.2)	21 (2.3)	113 (1.5)	
Multipara, *n* (%)	3594 (79.5)	2078 (72.8)	755 (82.0)	6394 (86.5)	<0.01
Han ethnicity, *n* (%)	4327 (95.7)	2603 (91.2)	882 (95.8)	7127 (96.4)	<0.01
Conception mode					<0.01
Natural conception	3586 (79.3)	2268 (79.5)	745 (80.9)	6039 (81.7)	
IVF or ICSI	618 (13.7)	291 (10.2)	118 (12.8)	967 (13.1)	
Others	50 (1.1)	43 (1.5)	10 (1.1)	78 (1.1)	
Missing	269 (5.9)	252 (8.8)	48 (5.2)	312 (4.2)	
Maternal education, *n* (%)					<0.01
Primary school or lower	50 (1.1)	19 (0.7)	4 (0.4)	47 (0.6)	
Junior high school	386 (8.5)	118 (4.1)	33 (3.6)	259 (3.5)	
High school or above	3986 (88.1)	2709 (94.9)	881 (95.7)	7063 (95.5)	
Missing	101 (2.2)	8 (0.3)	3 (0.3)	27 (0.4)	
Maternal alcohol consumption, *n* (%)	102 (2.3)	160 (5.6)	56 (6.1)	570 (7.7)	<0.01
Smoking, *n* (%)	73 (1.6)	60 (2.1)	8 (0.9)	115 (1.6)	0.06
South area, *n* (%)	3325 (73.5)	896 (31.4)	576 (62.5)	5611 (75.9)	<0.01

Abbreviations: FAS—Folic acid supplements; BMI—body mass index; IVF—in vitro fertilization; ICSI—intracytoplasmic sperm injection. ^†^ A chi-square test for categorical variables were used to examine the difference between the four groups.

**Table 2:** Incidences [N (%)] and aRRs (95% CIs) for pregnancy outcomes by FAS groups.

Outcomes	No-FAS		Peri-FAS		Mid-FAS		Late-FAS	
	N (%)	aRRs (95% CIs)	N (%)	aRRs (95% CIs)	N (%)	aRRs (95% CIs)	N (%)	aRRs (95% CIs)
GDM ^†^	1177 (26.0)	Reference	702 (24.6)	0.97 (0.86, 1.09)	2342 (28.2)	1.08 (0.99, 1.17)	/	/
GHDs ^†^	470 (10.4)	Reference	301 (10.6)	1.11 (0.95, 1.31)	736 (8.9)	0.84 (0.74, 0.96)	/	/
Preeclampsia ^†^	223 (4.9)	Reference	155 (5.4)	1.21 (0.97, 1.50)	336 (4.0)	0.81 (0.67, 0.97)	/	/
Preterm birth	591 (13.1)	Reference	310 (10.9)	0.90 (0.78, 1.05)	134 (14.6)	1.19 (0.97, 1.46)	694 (9.4)	0.67 (0.59, 0.76)
Macrosomia	792 (17.5)	Reference	433 (15.2)	0.89 (0.77, 1.02)	171 (18.6)	1.09 (0.91, 1.32)	1236 (16.7)	0.93 (0.84, 1.03)
SGA ^‡^	309 (8.1)	Reference	103 (5.5)	0.72 (0.57, 0.91)	44 (5.7)	0.72 (0.52, 1.01)	401 (6.0)	0.74 (0.63, 0.87)
LGA ^‡^	758 (19.8)	Reference	401 (21.6)	1.03 (0.89, 1.19)	158 (20.6)	1.05 (0.86, 1.27)	1191 (17.7)	0.88 (0.79, 0.97)

Abbreviations: FAS—Folic acid supplements; aRRs—adjusted relative risks; GDM—gestational diabetes mellitus; GHDs—gestational hypertensive disorders; SGA—small for gestational age; LGA—large for gestational age. ^†^ For GDM, GHDs and preeclampsia, which are diagnosed in mid-pregnancy, the late-FAS group was combined with the mid-FAS group for analysis. The analyses for GDM, GHDs, and preeclampsia in the mid-FAS group (which includes late-FAS participants for these mid-pregnancy outcomes) were based on 8317 participants. ^‡^ SGA and LGA analyses included 13,206 participants with complete birth weight and gestational week data. All adjusted RRs (95% CIs) were adjusted for maternal age, pre-pregnancy BMI, parity, ethnicity, conception mode, maternal education, pre-pregnancy smoking, and maternal alcohol consumption.

## Data Availability

The data described in the manuscript, codebook, and analytic code will not be made available because specific consent has not been obtained from either participants or ethics committee approving the study at the time of its conduct.

## Appendix A

**Table A1:** Characteristics of included and excluded participants.

Characteristics	Included (*n* = 15,694)	Excluded (*n* = 5336)
Maternal age, years, *n* (%)		
<35	3842 (24.5)	1394 (26.1)
35–39	9462 (60.3)	2945 (55.2)
40–44	2253 (14.4)	923 (17.3)
≥45	137 (0.9)	74 (1.4)
Pre-pregnancy BMI, *n* (%)		
Underweight	1347 (8.6)	265 (7.6)
Normal weight	11,763 (75.0)	2517 (72.3)
Overweight	2201 (14.0)	596 (17.1)
Obesity	383 (2.4)	103 (3.0)
Multipara, *n* (%)	12,821 (81.69)	2943 (77.82)
Han ethnicity, *n* (%)	14,939 (95.19)	4439 (95.81)
Conception mode		
Natural conception	12,638 (85.3)	3216 (84.8)
IVF or ICSI	1994 (13.5)	524 (13.8)
Others	181 (1.2)	54 (1.4)
Maternal education, *n* (%)		
Primary school or lower	120 (0.8)	45 (1.2)
Junior high school	796 (5.1)	239 (6.4)
High school or above	14,639 (94.1)	3445 (92.4)
Maternal alcohol consumption, *n* (%)	888 (5.7)	293 (5.5)
smoking, *n* (%)	256 (1.6)	59 (1.1)
south area, *n* (%)	10,408 (66.3)	3588 (67.2)

Abbreviations: BMI—Body mass index; IVF—in vitro fertilization; ICSI—intracytoplasmic sperm injection. Missing data was excluded for each variable except for maternal age, maternal alcohol consumption, smoking, and south area.

**Table A2:** Hematological indexes during late pregnancy.

Hematological Indexes	No-FAS	Peri-FAS	Mid-FAS	Late-FAS	*p*-Value ^†^
Hemoglobin (g/L)	117.6 (110.0 to 125.0)	117.4 (110.0 to 125.0)	118.8 (112.0 to 126.0)	120.6 (114.0 to 127.0)	<0.01
Red blood cell counts (×10^12^/L)	3.9 (3.6 to 4.1)	3.8 (3.6 to 4.1)	3.8 (3.6 to 4.1)	3.8 (3.6 to 4.1)	<0.01

Abbreviations: FAS—Folic acid supplements. ^†^ Kruskal–Wallis tests were used to examine the difference between the four groups.

**Table A3:** RRs (95% CIs) of univariate models for pregnancy outcomes.

Outcomes	No-FAS	Peri-FAS	Mid-FAS	Late-FAS
	RRs (95% CIs)	RRs (95% CIs)	RRs (95% CIs)	RRs (95% CIs)
GDM ^†^	Reference	0.93 (0.83, 1.03)	1.11 (1.03, 1.21)	/
GHDs ^†^	Reference	1.02 (0.87, 1.18)	0.84 (0.74, 0.95)	/
Preeclampsia ^†^	Reference	1.11 (0.90, 1.37)	0.81 (0.68, 0.97)	/
Preterm birth	Reference	0.81 (0.70, 0.94)	1.13 (0.93, 1.39)	0.69 (0.61, 0.77)
Macrosomia	Reference	0.84 (0.74, 0.96)	1.07 (0.89, 1.29)	0.95 (0.86, 1.04)
SGA ^‡^	Reference	0.66 (0.53, 0.83)	0.69 (0.50, 0.96)	0.72 (0.62, 0.84)
LGA ^‡^	Reference	1.11 (0.97, 1.27)	1.05 (0.86, 1.27)	0.87 (0.79, 0.96)

Abbreviations: FAS—Folic acid supplements; RRs—relative risks; GDM—gestational diabetes mellitus; GHDs—gestational hypertensive disorders; SGA—small for gestational age; LGA—large for gestational age. ^†^ For GDM, GHDs and preeclampsia, which are diagnosed in mid-pregnancy, the late-FAS group was combined with the mid-FAS group for analysis. The analyses for GDM, GHDs, and preeclampsia in the mid-FAS group (which includes late-FAS participants for these mid-pregnancy outcomes) were based on 8317 participants. ^‡^ SGA and LGA analyses included 13,206 participants with complete birth weight and gestational week data.

**Table A4:** Sensitivity analysis additionally adjusted for offspring sex.

Outcomes	No-FAS		Peri-FAS		Mid-FAS		Late-FAS	
	N (%)	aRRs (95% CIs)	N (%)	aRRs (95% CIs)	N (%)	aRRs (95% CIs)	N (%)	aRRs (95% CIs)
GDM ^†^	1177 (26.0)	Reference	702 (24.6)	0.95 (0.84, 1.07)	2342 (28.2)	1.09 (0.99, 1.18)	/	/
GHDs ^†^	470 (10.4)	Reference	301 (10.6)	1.14 (0.97, 1.34)	736 (8.9)	0.86 (0.75, 0.97)	/	/
Preeclampsia ^†^	223 (4.9)	Reference	155 (5.4)	1.12 (0.90, 1.40)	336 (4.0)	0.86 (0.72, 1.03)	/	/
Preterm birth	591 (13.1)	Reference	310 (10.9)	0.96 (0.82, 1.12)	134 (14.6)	1.22 (0.99, 1.50)	694 (9.4)	0.68 (0.60, 0.76)
Macrosomia	792 (17.5)	Reference	433 (15.2)	1.10 (0.96, 1.26)	171 (18.6)	1.10 (0.91, 1.33)	1236 (16.7)	0.85 (0.76, 0.94)
SGA ^‡^	309 (8.1)	Reference	103 (5.5)	0.68 (0.53, 0.89)	44 (5.7)	0.71 (0.51, 0.99)	401 (6.0)	0.74 (0.63, 0.87)
LGA ^‡^	758 (19.8)	Reference	401 (21.6)	1.04 (0.89, 1.20)	158 (20.6)	1.06 (0.87, 1.29)	1191 (17.7)	0.89 (0.80, 0.98)

Abbreviations: FAS—Folic acid supplements; aRRs—adjusted relative risks; GDM—gestational diabetes mellitus; GHDs—gestational hypertensive disorders; SGA—small for gestational age; LGA—large for gestational age. ^†^ For GDM, GHDs and preeclampsia, which are diagnosed in mid-pregnancy, the late-FAS group was combined with the mid-FAS group for analysis. The analyses for GDM, GHDs, and preeclampsia in the mid-FAS group (which includes late-FAS participants for these mid-pregnancy outcomes) were based on 8317 participants. ^‡^ SGA and LGA analyses included 13,206 participants with complete birth weight and gestational week data. All adjusted RRs (95% CIs) were adjusted for maternal age, pre-pregnancy BMI, parity, ethnicity, conception mode, maternal education, pre-pregnancy smoking, offspring sex, and maternal alcohol consumption.

**Table A5:** The interaction *p* for FAS length, maternal age, and pre-pregnancy BMI.

*p* for Interaction	GDM	Preeclampsia	GHDs	Preterm Birth	Macrosomia	SGA	LGA
Maternal age and FAS group	0.14	0.50	0.35	<0.01	0.03	0.63	0.33
Pre-pregnancy BMI and FAS group	0.14	0.01	<0.01	0.21	0.49	0.90	0.20
Maternal age, Pre-pregnancy BMI, and FAS group	0.26	0.59	0.83	0.51	0.66	0.99	0.50

Abbreviations: FAS—Folic acid supplements; GDM—gestational diabetes mellitus; GHDs—gestational hypertensive disorders; SGA—small for gestational age; LGA—large for gestational age.

**Table A6:** RRs (95% CIs) of univariate models stratified by maternal age.

Age	FAS Group	GDM ^†^	GHDs ^†^	Preeclampsia ^†^	Preterm Birth	Macrosomia	SGA ^‡^	LGA ^‡^
<35	no-FAS	Reference	Reference	Reference	Reference	Reference	Reference	Reference
	peri-FAS	0.92 (0.75, 1.13)	1.12 (0.82, 1.55)	0.97 (0.63, 1.51)	0.72 (0.55, 0.96)	1.16 (0.92, 1.47)	0.57 (0.37, 0.87)	1.35 (1.05, 1.73)
	mid-FAS	0.76 (0.62, 0.93)	0.59 (0.41, 0.85)	0.48 (0.28, 0.80)	0.51 (0.29, 0.88)	1.22 (0.84, 1.78)	0.63 (0.34, 1.19)	0.97 (0.66, 1.43)
	late-FAS	/	/	/	0.45 (0.32, 0.63)	1.09 (0.85, 1.38)	0.75 (0.52, 1.07)	0.80 (0.62, 1.02)
≥35	no-FAS	Reference	Reference	Reference	Reference	Reference	Reference	Reference
	peri-FAS	1.04 (0.91, 1.18)	1.12 (0.93, 1.34)	1.29 (1.01, 1.64)	0.93 (0.78, 1.11)	0.79 (0.67, 0.92)	0.73 (0.56, 0.96)	1.06 (0.89, 1.25)
	mid-FAS	1.11 (1.02, 1.22)	0.81 (0.71, 0.92)	0.81 (0.67, 0.98)	1.38 (1.10, 1.72)	1.05, (0.85, 1.30)	0.72 (0.49, 1.05)	1.10 (0.88, 1.38)
	late-FAS	/	/	/	0.69 (0.61, 0.78)	0.87 (0.78, 0.97)	0.71 (0.60, 0.85)	0.87 (0.78, 0.97)
	*p* for interaction	<0.01	0.12	0.09	<0.01	0.05	0.68	0.14

Abbreviations: FAS—Folic acid supplements; RRs—relative risks; GDM—gestational diabetes mellitus; GHDs—gestational hypertensive disorders; SGA—small for gestational age; LGA—large for gestational age. ^†^ For GDM, GHDs and preeclampsia, which are diagnosed in mid-pregnancy, the late-FAS group was combined with the mid-FAS group for analysis. The analyses for GDM, GHDs, and preeclampsia in the mid-FAS group (which includes late-FAS participants for these mid-pregnancy outcomes) were based on 8317 participants. ^‡^ SGA and LGA analyses included 13,206 participants with complete birth weight and gestational week data.

**Table A7:** RRs (95% CIs) of univariate models stratified by pre-pregnancy BMI.

Pre-Pregnancy BMI	FAS Group	GDM ^†^	GHDs ^†^	Preeclampsia ^†^	Preterm Birth	Macrosomia	SGA ^‡^	LGA ^‡^
Underweight	no-FAS	Reference	Reference	Reference	Reference	Reference	Reference	Reference
	peri-FAS	0.83 (0.54, 1.26)	0.17 (0.05, 0.58)	/	0.80 (0.47, 1.36)	0.64 (0.34, 1.18)	0.45 (0.22, 0.88)	0.68 (0.34, 1.33)
	mid-FAS	0.84 (0.62, 1.15)	0.65 (0.39, 1.08)	0.65 (0.31, 1.36)	0.80 (0.36, 1.77)	0.74 (0.30, 1.81)	0.76 (0.34, 1.69)	0.76 (0.31, 1.87)
	late-FAS	/	/	/	0.63 (0.42, 0.95)	0.93 (0.61, 1.41)	0.61 (0.41, 0.91)	0.85 (0.56, 1.31)
Normal	no-FAS	Reference	Reference	Reference	Reference	Reference	Reference	Reference
	peri-FAS	0.88 (0.77, 1.01)	0.94 (0.77, 1.15)	1.06 (0.80, 1.40)	0.73 (0.61, 0.87)	0.87 (0.75, 1.01)	0.72 (0.55, 0.94)	1.17 (0.99, 1.38)
	mid-FAS	1.12 (1.01, 1.23)	0.83 (0.71, 0.97)	0.87 (0.70, 1.08)	1.11 (0.88, 1.40)	1.04 (0.84, 1.28)	0.67 (0.46, 0.99)	1.01 (0.81, 1.27)
	late-FAS	/	/	/	0.65 (0.57, 0.74)	0.94 (0.84, 1.06)	0.74 (0.62, 0.89)	0.87 (0.77, 0.98)
Overweight/obesity	no-FAS	Reference	Reference	Reference	Reference	Reference	Reference	Reference
	peri-FAS	1.04 (0.83, 1.30)	1.21 (0.93, 1.57)	1.25 (0.89, 1.76)	1.10 (0.81, 1.48)	0.76 (0.58, 0.99)	0.68 (0.39, 1.19)	0.96 (0.72, 1.27)
	mid-FAS	1.39 (1.15, 1.67)	1.05 (0.84, 1.31)	0.85 (0.62, 1.16)	1.46 (0.91, 2.34)	1.44 (0.97, 2.14)	0.66 (0.26, 1.70)	1.41 (0.92, 2.17)
	late-FAS	/	/	/	0.95 (0.73, 1.24)	1.06 (0.85, 1.31)	0.67 (0.44, 1.04)	1.01 (0.81, 1.26)
	*p* for interaction	0.12	0.01	<0.01	0.17	0.39	0.78	0.20

Abbreviations: FAS—Folic acid supplements; RRs—relative risks; GDM—gestational diabetes mellitus; GHDs—gestational hypertensive disorders; SGA—small for gestational age; LGA—large for gestational age. ^†^ For GDM, GHDs and preeclampsia, which are diagnosed in mid-pregnancy, the late-FAS group was combined with the mid-FAS group for analysis. The analyses for GDM, GHDs, and preeclampsia in the mid-FAS group (which includes late-FAS participants for these mid-pregnancy outcomes) were based on 8317 participants. ^‡^ SGA and LGA analyses included 13,206 participants with complete birth weight and gestational week data.
